# Prevalence and Pattern of Refractive Errors Among Yanomami Indigenous People in the Brazilian Amazon: A Cross‐Sectional Observational Study

**DOI:** 10.1111/ceo.70143

**Published:** 2026-07-27

**Authors:** Maria Christina Chagas Ferreira, Marcos Antonio Pellegrini, Bianca Jorge Sequeira

**Affiliations:** ^1^ Universidade Federal de Roraima PROCISA Boa Vista Roraima Brazil

**Keywords:** health inequity, indigenous peoples, myopia, refractive errors, uncorrected refractive error, visual impairment

## Abstract

**Background:**

Refractive errors are the leading cause of preventable visual impairment worldwide, yet data from isolated Indigenous populations remain virtually absent from the global literature. The Yanomami, one of the largest Indigenous peoples in the Americas with recent and limited contact with non‐Indigenous society, have no prior epidemiological data on refractive errors

**Methods:**

A cross‐sectional observational study was conducted in 2024 at the Yanomami Indigenous Health House, Boa Vista, Roraima, Brazil. A total of 158 self‐identified Yanomami individuals aged 5 years or older were examined by an ophthalmologist. Refractive status was classified according to International Myopia Institute criteria.

**Results:**

Emmetropia was observed in 67.7% of participants, declining markedly with age from 100% in children aged 5 to 9 years to 38.6% in those aged 40 to 59 years. Myopia was present in 16.5%, all low myopia, absent in children under 10 years, with no high myopia identified. Astigmatism affected 24.1% and hyperopia 12.7%. Presbyopia was identified in 24.7% of participants and 52.0% of those aged 35 years or older. Overall, 25.3% presented with reduced visual acuity, of whom 67.5% improved to normal range (20/30 or better) (*p* < 0.001).

**Conclusions:**

This first characterisation of the Yanomami refractive profile reveals a distinct myopia pattern shaped by high outdoor exposure and minimal near‐work demands. Yet refractive correction remains effectively inaccessible, leaving preventable visual impairment unaddressed and reflecting a profound health inequity. Corrective lens provision is a high‐impact, scalable intervention for this underserved community.

## 1 Introduction

Refractive errors are the leading cause of visual impairment worldwide [1] and one of the most preventable contributors to functional vision loss across all age groups [2]. According to global estimates, uncorrected refractive error accounts for a substantial proportion of moderate and severe visual impairment, with significant consequences for education, productivity, social participation, and quality of life [1]. Despite the simplicity and cost‐effectiveness of refractive correction, access to vision assessment and corrective lenses remains highly unequal, disproportionately affecting populations living in remote, low‐resource, and underserved settings.

Indigenous peoples experience persistent health inequities driven by geographic isolation, socioeconomic vulnerability, and limited access to culturally appropriate health services [3, 4, 5]. In the Americas, these disparities are particularly pronounced in remote regions of the Amazon, where logistical barriers, workforce shortages, and linguistic diversity restrict the availability of specialised eye care [6]. As a result, visual impairment due to uncorrected refractive error often remains undiagnosed and untreated, even when effective interventions are readily available [7, 8].

Evidence on refractive errors in Indigenous populations is scarce and heterogeneous [9, 10]. Existing studies have largely focused on urban or semi‐urban Indigenous groups or have addressed specific ocular conditions rather than population‐level refractive profiles [9]. Data from Indigenous peoples living in areas of recent contact with non‐Indigenous society are especially limited, constraining the development of targeted public health strategies. Environmental, cultural, and lifestyle factors, including high levels of outdoor activity, limited formal education, and reduced exposure to prolonged near work, suggest that the distribution and pattern of refractive errors in these populations may differ from those observed in urban settings, underscoring the need for context‐specific epidemiological data.

The Yanomami are one of the largest Indigenous groups in the Brazilian Amazon and are characterised by high territorial mobility, geographic isolation, and a history of relatively recent and limited contact with non‐Indigenous society. Eye health data in this population are particularly scarce. While previous studies have documented a high burden of visual impairment and blindness among the Yanomami, uncorrected refractive error has consistently emerged as a leading and potentially avoidable cause of vision loss [11]. However, detailed information on the prevalence, type, and distribution of refractive errors in this population remains lacking.

This study aimed to estimate the prevalence and pattern of refractive errors among Yanomami Indigenous people living in the Brazilian Amazon and to assess the functional impact of refractive correction on visual acuity.

## 2 Methods

### 2.1 Study Design and Setting

This was a cross‐sectional, observational study conducted between June and August 2024 at the Yanomami Indigenous Health House (Casa de Saúde Indígena Yanomami, CASAI‐Y), located in Boa Vista, Roraima, Brazil. CASAI‐Y is a regional referral and accommodation centre within the Yanomami Special Indigenous Health District (DSEI‐Y), hosting Indigenous individuals from remote communities across the Yanomami Indigenous Territory who travel to Boa Vista for outpatient health services unavailable at primary care units. Owing to geographic, legal, and logistical constraints that preclude population‐based surveys within the Yanomami Indigenous Territory, CASAI‐Y represents the most feasible setting to access a diverse and demographically representative cross‐section of this highly mobile Indigenous population [12, 13].

The study included self‐identified Yanomami Indigenous individuals aged 5 years or older who were present at CASAI‐Y during the data collection period. Exclusion criteria included refusal to participate, inability to complete the ophthalmological examination due to cognitive impairment or insufficient clinical conditions, or refusal of any component of the evaluation.

Participation was voluntary and based on informed consent; for minors, consent was obtained from a parent or legal guardian, with assent from the child when appropriate. All informed consent and assent documents were translated into the participants' native language and explained orally by a native‐speaking Indigenous interpreter. Given the high prevalence of illiteracy among participants, the consent process was audio recorded to ensure full ethical compliance and participant comprehension. In cases in which written consent could not be provided, the interpreter signed the consent form on the participant's behalf, in accordance with the approved protocol.

### 2.2 Study Population and Sample

At the time of the study, 278 eligible individuals aged 5 years or older, belonging to four officially registered Yanomami subgroups (Yanomami, Sanuma, Xirixana, and Xiriana), were present at the facility. A total of 158 individuals met the inclusion criteria and agreed to participate, corresponding to 56.8% of the eligible population. The participant flow is presented in Figure 1.

**FIGURE 1:**
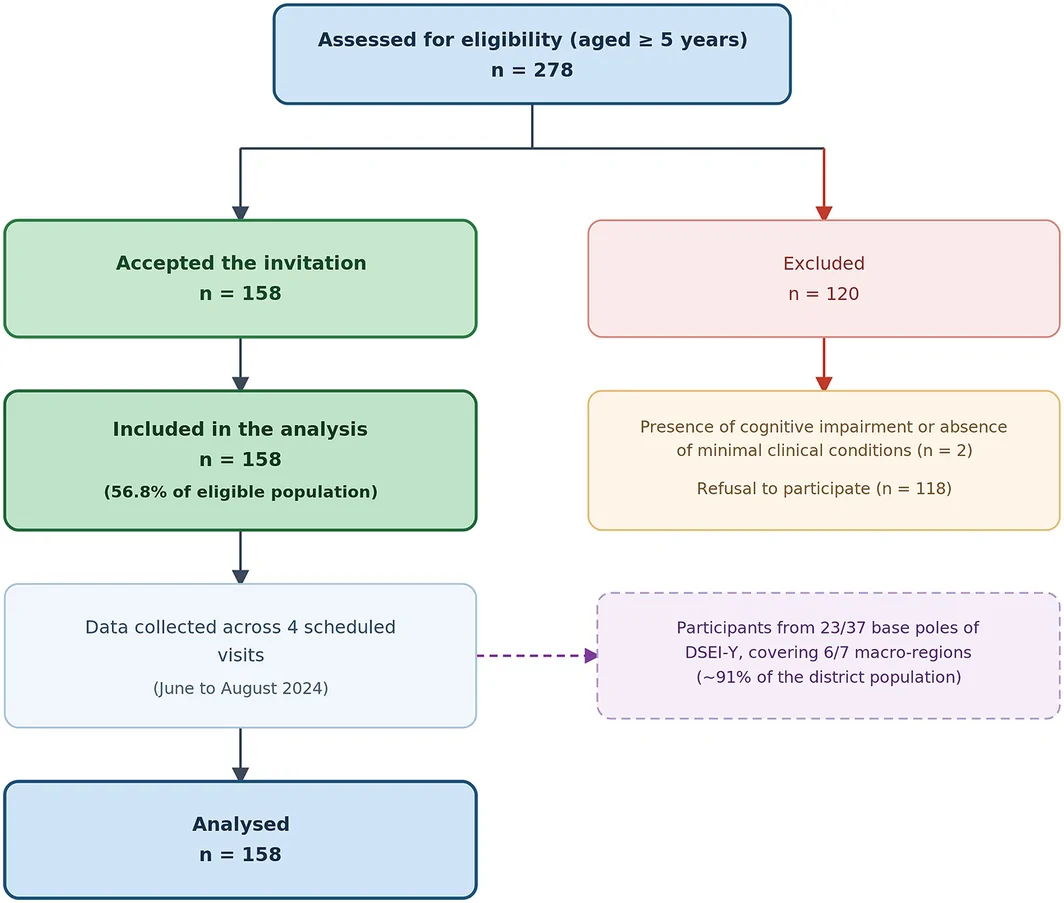
Flow diagram showing recruitment and inclusion of participants in the study (2024).

The sample size was not predefined by probabilistic sampling; rather, it was based on the total accessible population during the study period. Nevertheless, the achieved sample exceeded the minimum estimated size required to obtain a 95% confidence level with a 5% margin of error in a homogeneous population.

Participants originated from 23 of the 37 base poles of the DSEI‐Y, covering six of the seven macro‐regions of the Yanomami territory and representing approximately 91% of the district population. A moderate positive correlation (Pearson's *r* = 0.57) was observed between the number of examined individuals per base pole and the total population size of each locality, suggesting proportional geographic representation. Given the non‐random nature of recruitment and the facility‐based design, potential selection bias cannot be excluded; however, the proportional geographic distribution observed supports partial representativeness of the broader DSEI‐Y population.

### 2.3 Data Collection and Ophthalmological Examination

Data collection occurred during four scheduled field visits and was organised according to a structured, three‐step ophthalmological assessment protocol. In the initial step, participants' demographic information (sex, age, community of origin, and reference health post) was recorded, and informed consent or assent procedures were completed.

The second step consisted of objective ophthalmological measurements performed by trained personnel. Refractive screening was conducted using a Welch Allyn Spot Vision Screener handheld photoscreener. Intraocular pressure was measured with a Huvitz HNT‐7000 non‐contact tonometer, and retinal images were obtained using an Eyer portable fundus camera to document posterior segment findings and to rule out ocular conditions that could influence refractive assessment.

Immediately after the objective measurements, the third step involved a clinical interview to collect self‐reported ocular history and general health information. This was followed by slit‐lamp biomicroscopy performed with a portable slit lamp and subjective refraction carried out by an ophthalmologist. Both objective refractive screening and subjective refraction were performed without cycloplegia.

Distance visual acuity was measured monocularly and without correction using a Snellen “E” chart, as recommended by the Brazilian Ministry of Health, and a culturally adapted pictorial chart specifically developed for the Yanomami population. Both charts were positioned at a distance of three meters from the participant, in an adequately illuminated environment. For participants with presenting visual acuity worse than 20/30 or who reported visual difficulties, best‐corrected visual acuity was assessed dynamically using a Greens refractor.

Near visual acuity was evaluated in participants aged 35 years or older using a Jaeger near vision card with the “E” optotype and a corresponding pictorial version adapted for this study. Testing was performed at a standard distance of 37 cm, and near addition was determined using the Greens refractor when required.

Throughout all interviews and examinations, Indigenous interpreters fluent in the participant's specific dialect facilitated communication. Pharmacological pupillary dilation with tropicamide was used only in selected older participants, exclusively to enable fundoscopic examination, and was performed only after refraction had been completed; no adverse events were observed. At the end of the assessment, prescriptions for corrective lenses were issued when indicated, and participants requiring further care were referred through the Brazilian Unified Health System (SUS).

### 2.4 Definitions and Outcomes

Refractive status was classified according to the International Myopia Institute (IMI) standards [14]. Spherical equivalent (SE) was calculated for each eye. Myopia was defined as SE ≤ −0.50 D, hyperopia as SE ≥ +0.50 D, and astigmatism as absolute cylinder ≥ 1.00 D in at least one eye. In a sensitivity analysis, emmetropia was additionally classified using a multicomponent functional criterion [15], which requires an absolute SE < 0.50 D together with an astigmatism magnitude ≤ 0.75 D in both eyes, in order to account for the visual impact of astigmatism not captured by the spherical equivalent alone. Combined refractive errors were further categorised as compound myopic astigmatism, compound hyperopic astigmatism, and simple astigmatism. Presbyopia was defined as the need for near addition ≥ +1.00 D to achieve functional near vision at 37 cm (J1 level). This corresponds to functional presbyopia, which may differ from strictly physiological definitions based on the amplitude of accommodation. Uncorrected refractive error was defined as presenting visual acuity worse than 20/30 that improved to 20/30 or better after subjective refraction.

The primary outcome was the prevalence of refractive errors among Yanomami Indigenous individuals. Secondary outcomes included the distribution of refractive error types and the change in visual acuity following refractive correction.

### 2.5 Statistical Analysis

Continuous variables were summarised as means and standard deviations; categorical variables were expressed as absolute frequencies and percentages. Prevalence estimates were reported with stratification by sex, age group, and Yanomami subethnic subgroup. Age‐standardised prevalence estimates were calculated by the direct method. Visual acuity measured with the Snellen “E” and culturally adapted pictorial charts was converted to decimal notation to allow calculation of means and paired comparisons. Comparisons between presenting and best‐corrected visual acuity were performed using paired‐sample Student's *t*‐tests. Associations between refractive errors and demographic variables were evaluated using chi‐squared tests or Fisher's exact tests, as appropriate. Odds ratios (ORs) with 95% confidence intervals were estimated by binary logistic regression for age‐related trends, using age as a continuous predictor, and as crude odds ratios from 2 × 2 contingency tables for associations between refractive errors and ocular conditions such as pterygium and cataract, with statistical significance assessed by Fisher's exact test. All statistical tests were two‐sided; a *p* value of less than 0.05 was considered statistically significant. Analyses were performed using Microsoft Excel, Statistica version 12 (TIBCO Software Inc.), and StatPlus (AnalystSoft). Given the convenience sampling approach, analyses were interpreted descriptively, emphasising epidemiological patterns rather than causal inference.

### 2.6 Ethics Statement

The study was approved by the Research Ethics Committee of the Federal University of Roraima and the Brazilian National Research Ethics Commission (Approval No. 6.479.368‐CEP/CONEP). All procedures adhered to the tenets of the Declaration of Helsinki.

## 3 Results

### 3.1 Participant Characteristics

A total of 158 Yanomami individuals aged 5 years or older were included in the analysis, representing 56.8% of the eligible population present at CASAI‐Y during the study period. Participants belonged to four officially registered Yanomami subethnic subgroups: Yanomami (65.8%), Sanuma (24.1%), Xirixana (7.0%), and Xiriana (3.2%), and originated from multiple regions within the DSEI‐Y. The final sample comprised 102 males (64.6%) and 56 females (35.4%), with a mean age of 32.4 years (SD 13.8). Demographic characteristics are presented in Table 1.

**TABLE 1:** Demographic characteristics of participants (*N* = 158).

	Male (*n* = 102)	Female (*n* = 56)	Total (*N* = 158)
Mean age (SD), years	33.5 (13.7)	30.4 (13.9)	32.4 (13.8)
Age group, *n* (%)			
0–19	19 (18.6)	13 (23.2)	32 (20.3)
20–39	40 (39.2)	27 (48.2)	67 (42.4)
40–59	42 (41.2)	15 (26.8)	57 (36.1)
60–80	1 (1.0)	1 (1.8)	2 (1.3)
Subethnic subgroup, *n* (%)			
Yanomami	62 (60.8)	42 (75.0)	104 (65.8)
Sanuma	31 (30.4)	7 (12.5)	38 (24.1)
Xirixana	5 (4.9)	6 (10.7)	11 (7.0)
Xiriana	4 (3.9)	1 (1.8)	5 (3.2)

*Note:* Data are *n* (%) unless otherwise stated.

Abbreviation: SD = standard deviation.

### 3.2 Prevalence and Pattern of Refractive Errors

Among the 158 participants, strict emmetropia was observed in 107 individuals (67.7%). A clear age‐related decline was evident, from 100% in children aged 5–9 years (11/11) to 95.2% in adolescents aged 10–19 years (20/21), 80.6% in adults aged 20–39 years (54/67), and 38.6% in those aged 40–59 years (22/57). No emmetropic individuals were identified among participants aged 60 years or older.

Myopia was present in 16.5% of participants (26/158); all cases were low myopia and no high myopia (SE ≤ −6.00 D) was identified. Notably, myopia was completely absent in children aged 5–9 years (0/11; 0.0%). Prevalence increased progressively with age, from 4.8% in adolescents aged 10–19 years to 17.9% in adults aged 20–39 years and 21.1% in those aged 40–59 years. Sex distribution was similar, with 16.7% in males and 16.1% in females.

Astigmatism was the most prevalent refractive condition, affecting 24.1% of participants (38/158), and was predominantly observed in combination with spherical refractive errors. Hyperopia was present in 12.7% (20/158), with all cases classified as mild. Among participants aged 35 years or older (*n* = 75), presbyopia was identified in 39 individuals (52.0%), with a mean near addition of +2.04 D. Notably, 10 of the 39 presbyopic individuals (25.6%) were strict emmetropes who required near addition without distance correction. The full prevalence and classification of refractive errors are presented in Table 2.

**TABLE 2:** Prevalence and classification of refractive errors according to IMI criteria (*N* = 158).

Classification	*n*	%	Age‐std %	OR/year (95% CI); *p*
Emmetropia (SE −0.50 to +0.50 D)	107	67.7	67.7	0.93 (0.91–0.95); < 0.001
Any refractive error	51	32.3	36.1	—
Myopia (SE ≤ −0.50 D)	26	16.5	15.9	1.045 (1.010–1.081); 0.011
Low myopia only	26	16.5	—	—
High myopia (SE ≤ −6.00 D)	0	0.0	—	—
Hyperopia (SE ≥ +0.50 D)	20	12.7	—	1.205 (1.113–1.304); < 0.001
Mild hyperopia only	20	12.7	—	—
Astigmatism (cylinder ≥ 1.00 D)	38	24.1	30.7	1.101 (1.059–1.144); < 0.001
Compound myopic astigmatism	20	12.7	—	—
Compound hyperopic astigmatism	13	8.2	—	—
Simple astigmatism	5	3.2	—	—
Presbyopia (age ≥ 35 years; *n* = 75)	39	52.0	—	*r* = 0.355; *p* = 0.002
Reduced presenting VA	40	25.3	—	*t* = 7.84; *p* < 0.001

*Note:* Age‐standardised estimates by direct standardisation. Refractive categories not mutually exclusive except strict emmetropia. All cylinder values negative. Age ≥ 60 years (*n* = 2; 1.3%) is shown for descriptive purposes only; inferential age analyses modelled age as a continuous variable.

Abbreviations: CI = confidence interval; D = dioptre; OR = odds ratio; RE = refractive error; SE = spherical equivalent; VA = visual acuity.

### 3.3 Age‐Related Associations and Regression Analysis

Binary logistic regression confirmed significant age‐related associations for all three principal refractive errors. The odds of myopia increased by 4.5% per year of age (OR 1.045; 95% CI 1.010–1.081; *p* = 0.011). Hyperopia showed the strongest age‐related effect (OR 1.205; 95% CI 1.113–1.304; *p < 0.001*), largely reflecting its concentration in adults aged 40 years and older. Astigmatism also increased significantly with age (OR 1.101; 95% CI 1.059–1.144; *p* < 0.001). No significant associations were observed with sex or subethnic subgroup for any refractive error. Full regression results are presented in Table 3.

**TABLE 3:** Association between age and refractive errors: Binary logistic regression (*N* = 158).

Refractive error	β (SE)	OR per year	95% CI	*p* value
Myopia	0.044 (0.017)	1.045	1.010–1.081	0.011
Hyperopia	0.186 (0.041)	1.205	1.113–1.304	< 0.001
Astigmatism	0.096 (0.020)	1.101	1.059–1.144	< 0.001

*Note:* All models include age as continuous predictor. *p* values from Wald statistics.

Abbreviations: CI = confidence interval; OR = odds ratio; SE = standard error.

### 3.4 Uncorrected Refractive Error and Visual Acuity

A total of 40 participants (25.3%) presented with reduced distance visual acuity (presenting visual acuity worse than 20/30). In 27 of them (67.5%), acuity returned to the normal range (20/30 or better) with optical correction, identifying uncorrected refractive error as the cause; the improvement after correction was statistically significant (paired *t*‐test, *p* < 0.001). In the remaining 13 cases (32.5%), acuity did not return to normal with correction, indicating non‐refractive causes such as cataract or other ocular conditions.

Trends toward association were observed between pterygium (8.2%) and astigmatism (OR 2.22; 95% CI 0.60–8.18; *p* = 0.226), and between cataract (7.6%) and astigmatism (OR 3.57; 95% CI 0.92–13.86; *p* = 0.066), though neither reached statistical significance, likely owing to the small size of the affected subgroups. No association was observed between cataract and myopia (OR 0.68; 95% CI 0.08–5.75; *p* = 0.625). These findings are summarised in Table 4.

**TABLE 4:** Associations between comorbid ocular conditions and refractive errors.

Condition (prevalence)	Outcome	OR (95% CI)	*p* value	Interpretation
Pterygium (8.2%; *n* = 13)	Astigmatism	2.22 (0.60–8.18)	0.226	Trend; underpowered
Cataract (7.6%; *n* = 12)	Astigmatism	3.57 (0.92–13.86)	0.066	Borderline
Cataract (7.6%; *n* = 12)	Myopia	0.68 (0.08–5.75)	0.625	No association

*Note:* Fisher exact test used throughout. Analyses are hypothesis‐generating.

Abbreviations: CI = confidence interval; OR = odds ratio.

## 4 Discussion

Recent data from a 2025 study on Yanomami visual status reported a prevalence of moderate or worse visual impairment and blindness of 15.8% based on presenting visual acuity, decreasing to 8.2% with best‐corrected visual acuity, with cataract and uncorrected refractive error as the leading causes [11]. In that analysis, uncorrected ametropias accounted for 74.3% of initial visual impairment, and significant visual acuity gains were observed after refractive correction. The present article provides the first detailed characterisation of the Yanomami refractive profile, expanding those findings to describe distribution by type, magnitude, age, sex, and subethnic subgroup, and associations of pterygium and cataract with the presence of astigmatism.

The prevalence of strict emmetropia in the Yanomami population was high at 67.7%, consistent with a remote, low near‐work environment. This estimate was identical under the multicomponent functional criterion [15], as no strictly emmetropic participant exhibited astigmatism above 0.75 D. Yanomami emmetropia was higher than in Indigenous Brazilians from Avai/SP (approximately 45%) [16], rural South Indian adults aged 40 years or older (approximately 50%) [17, 18], and substantially higher than in the Barbados Eye Study (approximately 31% in adults aged 40–84 years) [19] and very elderly European cohorts [7]. Rural Southeast Asian populations showed comparable rates (approximately 65%) [20]. These differences likely reflect contrasting degrees of urbanisation, age structure, and environmental exposure.

The Yanomami pattern of high emmetropia in younger age groups aligns with evidence for a protective role of sustained outdoor exposure and minimal near‐work demands during childhood. Meta‐analytic data demonstrate a dose–response relationship between time outdoors and reduced myopia incidence [21]. Consistent with this, myopia was completely absent in children aged 5–9 years in the present study. However, intense lifelong solar exposure may have a dual effect: while high ambient light levels protect against developmental myopia through dopamine‐mediated mechanisms [21], cumulative ultraviolet exposure may accelerate age‐related lenticular changes [22, 23], partially explaining the marked emmetropia decline observed after mid‐life in this cohort.

The overall myopia prevalence of 16.5% was lower than in a Brazilian population sample (25.3%, peaking near 42% in the mid‐twenties) [24], and consistent with the low myopia previously reported in Amazonian Indigenous people [8], as well as in rural Australian Aboriginal populations (11%–15%) [25], likely reflecting the protective effects of extensive outdoor activity and low near‐work exposure. This contrast was most striking in the younger age groups: myopia was absent in children aged 5–9 years and reached only 4.8% in those aged 10–19 years. In schoolchildren from other low‐latitude regions, by contrast, myopia prevalence has been reported at 20.4% in north‐eastern Brazil [26] and at 15.7% in urban and 9.2% in rural Colombian children [27], despite similarly high ambient light exposure. The near‐complete absence of myopia in Yanomami children, together with the absence of any high myopia across the sample, supports a predominant role of environmental factors, particularly minimal near‐work demand, in this population.

Astigmatism affected 24.1% of participants, a higher prevalence than the 15.5% reported among Indigenous people of the northwestern Amazon, under the same threshold [8], the closest available comparison for an Amazonian Indigenous population. This difference may reflect the broader age range of the present sample, since astigmatism increased significantly with age (OR 1.101 per year; *p* < 0.001), as well as methodological differences in refraction technique and in the unit of analysis. Although most astigmatism in this population was of low magnitude, higher‐magnitude cylinders were also present, reaching up to 5.00 D; these higher values occurred exclusively among participants already classified as ametropic, which is consistent with the concordance between the strict and functional emmetropia estimates. The overall pattern is compatible with changes associated with chronic ultraviolet exposure at tropical latitudes.

Hyperopia showed a strong age‐related increase (OR 1.205 per year; *p* < 0.001), largely concentrated in adults aged 40 years and older, consistent with age‐related lenticular shifts. Presbyopia affected 24.7% of participants overall and 52.0% of those aged 35 years or older, confirming the substantial unmet need for near vision correction in this population. Comparable presbyopia data for other Amazonian Indigenous groups are lacking, and future studies should address this gap.

Trends toward associations between pterygium and astigmatism, and between cataract and astigmatism, did not reach statistical significance, likely due to the small size of the affected subgroups. These hypothesis‐generating findings warrant investigation in larger samples and align with the 2025 study's identification of cataract as a key cause of persistent visual impairment after refractive correction [11].

The findings have direct implications for eye health planning in the Yanomami territory. The high proportion of participants (25.3%) with reduced visual acuity, largely correctable with refraction, and the substantial improvement observed with optical correction (67.5%; *p* < 0.001) underscore the potential impact of spectacle provision programmes at CASAI‐Y and in remote communities. Culturally adapted assessment tools, such as the pictogram charts used in this study, and teleophthalmology services could address the logistical barriers inherent to this setting.

### 4.1 Strengths and Limitations

This study has several strengths. It provides the first systematic characterisation of the refractive profile of the Yanomami, a population with recent and limited contact with non‐Indigenous society. Although recruitment relied on an opportunity sample at CASAI‐Y, the achieved sample exceeded the minimum size required for a 95% confidence level with a 5% margin of error and drew on participants from 23 of the 37 base poles of the DSEI‐Y, covering six of the seven macro‐regions and representing approximately 91% of the district population, with a moderate positive correlation between the number of individuals examined per base pole and local population size (Pearson's *r* = 0.57). This convergence indicates that a facility‐based design at a referral health house can yield a geographically representative cross‐section of a highly mobile population, offering a feasible and replicable strategy for studying remote, under‐researched groups in whom territory‐wide population surveys are not logistically possible. Refractive status was classified by an ophthalmologist according to standardised IMI criteria, and culturally adapted pictorial optotypes were used to ensure valid testing in a largely non‐literate population.

Some limitations should also be acknowledged. The facility‐based design at CASAI‐Y may introduce selection bias. However, as a referral and accommodation centre rather than a hospital, CASAI‐Y hosts individuals undergoing outpatient treatment together with their accompanying family members, the latter generally being healthy community members. The sample was therefore not concentrated among severely ill patients, and refraction relied primarily on objective autorefraction, limiting the influence of acute illness. Participants who were not in adequate clinical condition to undergo the examination were excluded, as specified in the eligibility criteria. A previous analysis of this population showed that attendance at CASAI‐Y reflected the regional distribution of the Yanomami, supporting the representativeness of the sample [11]. In addition, small subgroup sizes limited the statistical power to detect associations with comorbid ocular conditions, and cultural factors, including lower participation among women and older individuals, may have influenced the demographic composition of the sample.

A further limitation concerns refractive measurement, as refraction was performed without cycloplegia. In principle, this could introduce accommodative pseudomyopia and thus overestimate myopia and underestimate hyperopia in younger participants. This concern is attenuated on two grounds. First, such a bias would act to inflate myopia, so the low myopia prevalence observed (16.5%, with no high myopia and complete absence in children aged 5 to 9 years) is unlikely to be artefactual and, if anything, conservative. Second, accommodative capacity, preserved in younger individuals, declines progressively from the mid‐forties with the onset of presbyopia, so the potential for an accommodative shift is substantially smaller in middle‐aged and older participants [28]. The main residual caveat is therefore a possible modest underestimation of hyperopia in young adults.

Future research should incorporate cycloplegic refraction for this purpose and employ longitudinal and territory‐wide designs to clarify how environmental and lifestyle factors shape refractive development in this population.

### 4.2 Conclusions

This study provides the first characterisation of the refractive profile of the Yanomami people. The population demonstrates a distinct refractive pattern consistent with a lifestyle shielded from urban risk factors, with no myopia in children under 10 years of age and no high myopia detected. Despite this, refractive correction remains effectively inaccessible, leaving preventable visual impairment unaddressed and reflecting a profound health inequity. Corrective lens provision and culturally adapted eye care services represent high‐impact, scalable interventions for this underserved community.

## Contributors

**Maria Christina Chagas Ferreira:** conceptualisation, data curation, formal analysis, investigation, methodology, project administration, resources, writing (original draft and review and editing), and final responsibility for the decision to submit for publication. **Marcos Antonio Pellegrini:** conceptualisation, methodology, resources, supervision. **Bianca Jorge Sequeira:** formal analysis, methodology, supervision, writing (original draft and review and editing).

## Funding

The authors have nothing to report.

## Conflicts of Interest

The authors declare no conflicts of interest.

## Data Availability

The data that support the findings of this study are available on request from the corresponding author. The data are not publicly available due to privacy or ethical restrictions. As this study involves Indigenous populations, deidentified data access requires approval by the relevant Indigenous community leadership, ensuring respect for Indigenous data governance.
